# Accelerated development of cerebral small vessel disease in young stroke patients

**DOI:** 10.1212/WNL.0000000000003123

**Published:** 2016-09-20

**Authors:** Renate M. Arntz, Steffen M.A. van den Broek, Inge W.M. van Uden, Mohsen Ghafoorian, Bram Platel, Loes C.A. Rutten-Jacobs, Noortje A.M. Maaijwee, Pauline Schaapsmeerders, Hennie C. Schoonderwaldt, Ewoud J. van Dijk, Frank-Erik de Leeuw

**Affiliations:** From Donders Institute for Brain, Cognition and Behaviour, Department of Neurology (R.M.A., S.M.A.v.d.B., I.W.M.v.U., L.C.A.R.-J., N.A.M.M., P.S., H.C.S., E.J.v.D., F.-E.d.L.), and Diagnostic Image Analysis Group, Department of Radiology and Nuclear Medicine (M.G., B.P.), Radboudumc; Institute for Computing and Information Sciences (M.G.), Radboud University, Nijmegen, the Netherlands; and Department of Clinical Neurosciences, Neurology Unit (L.C.A.R.-J.), University of Cambridge, UK.

## Abstract

**Objective:**

To study the long-term prevalence of small vessel disease after young stroke and to compare this to healthy controls.

**Methods:**

This prospective cohort study comprises 337 patients with an ischemic stroke or TIA, aged 18–50 years, without a history of TIA or stroke. In addition, 90 age- and sex-matched controls were included. At follow-up, lacunes, microbleeds, and white matter hyperintensity (WMH) volume were assessed using MRI. To investigate the relation between risk factors and small vessel disease, logistic and linear regression were used.

**Results:**

After mean follow-up of 9.9 (SD 8.1) years, 337 patients were included (227 with an ischemic stroke and 110 with a TIA). Mean age of patients was 49.8 years (SD 10.3) and 45.4% were men; for controls, mean age was 49.4 years (SD 11.9) and 45.6% were men. Compared with controls, patients more often had at least 1 lacune (24.0% vs 4.5%, *p* < 0.0001). In addition, they had a higher WMH volume (median 1.5 mL [interquartile range (IQR) 0.5–3.7] vs 0.4 mL [IQR 0.0–1.0], *p* < 0.001). Compared with controls, patients had the same volume WMHs on average 10–20 years earlier. In the patient group, age at stroke (β = 0.03, 95% confidence interval [CI] 0.02–0.04) hypertension (β = 0.22, 95% CI 0.04–0.39), and smoking (β = 0.18, 95% CI 0.01–0.34) at baseline were associated with WMH volume.

**Conclusions:**

Patients with a young stroke have a higher burden of small vessel disease than controls adjusted for confounders. Cerebral aging seems accelerated by 10–20 years in these patients, which may suggest an increased vulnerability to vascular risk factors.

Incidence of stroke at young age is increasing,^1^ which has been explained by a high prevalence of traditional vascular risk factors.^2^ However, the prevalence of these risk factors is not only high in (young) stroke patients, but often equally high in the general population.^3,4^ Accordingly, most young individuals with these risk factors never experience a stroke. This may suggest that patients who do develop a stroke are more vulnerable to risk factors than those who do not. Consequently, they may also have a higher risk of developing other (cerebro)vascular diseases. A possible marker of the brain's vulnerability to vascular risk factors is cerebral small vessel disease (SVD). In the elderly, SVD has convincingly been related to vascular risk factors and accelerated cognitive and motor decline.^5–7^

The prevalence of the full spectrum of SVD (lacunes, microbleeds, and white matter hyperintensity [WMH] volume) at variable intervals after stroke at young age, and its comparison with controls, has never been investigated. In addition, it is unknown whether the same risk factors in elderly are associated with SVD in patients with a stroke at young age. We hypothesized that SVD as a marker of early aging would be more frequent after stroke in young adults, compared with controls.

The aim of this study was to examine prevalence and risk factors of SVD as a marker of the brain's vulnerability to vascular risk factors in patients with a first-ever TIA or ischemic stroke aged 18–50 years after long-term follow-up and to compare this with controls.

## METHODS

### Study population.

This study is part of the Follow-Up of Transient Ischemic Attack and Stroke Patients and Unelucidated Risk Factor Evaluation (FUTURE) study, a prospective cohort study that investigates causes and consequences of a young stroke.^8^ We used the same methodology as the one employed in previous studies.^8^

The FUTURE study comprises all consecutive patients with a TIA or ischemic stroke, aged 18–50 years, admitted to the Radboud University Nijmegen Medical Centre from 1980 to 2010.^8^ For the definition of stroke and TIA, the WHO definition was used,^9,10^ in which stroke was defined as a rapidly evolving focal neurologic deficit, with no other than vascular cause lasting more than 24 hours.^8^ For TIA, the same definition was used, but lasting less than 24 hours.^8^ Exclusion criteria were cerebral venous sinus thrombosis and retinal infarction.^8^

Patients were identified through a prospective registry with a standardized collection of baseline and clinical characteristics and all patients underwent neurologic examination and brain imaging at the time of their index event.^8^ The assessment of stroke etiology (Trial of Org 10172 in Acute Stroke Treatment [TOAST])^11^ and severity (NIH Stroke Scale^12^) was done for all cases retrospectively by a validated approach,^13,14^ as these scales did not exist at the time when a substantial proportion of our patients experienced their qualifying event.^8^

### Standard protocol approvals, registrations, and patient consents.

The Medical Review Ethics Committee region Arnhem-Nijmegen approved the study. Written informed consent was obtained from all participants.

### Controls.

At follow-up, stroke-free controls were recruited among patients' spouses, relatives, or social environment. They had to be at least 18 years old without a clinical history of TIA or stroke. The control group and patient group were matched for age, sex, and level of education.^8^

### Follow-up.

Alive patients were approached by telephone and follow-up assessment was performed between 2009 and 2012. Subsequently, patients were given the opportunity to participate in a substudy in which they were invited to our research center for an extensive in-person follow-up examination, including physical examination and an extensive MRI protocol.^8^ Patients with MRI contraindications or known claustrophobia were excluded from the present study.

### MRI scanning and processing.

MRI scanning was performed on a 1.5T Magnetom scanner (Siemens, Erlangen, Germany). The scanning protocol included whole-brain 3D T1 magnetization-prepared rapid gradient echo sequence (repetition time [TR]/echo time [TE]/inversion time [TI] 2,730/2.95/1,000 ms; flip angle 7°; voxel size 1.0 × 1.0 × 1.0 mm); fluid-attenuated inversion recovery (FLAIR) pulse sequences (TR/TE/TI 12,220/85/2,200 ms; voxel size 1.0 × 1.2 × 3.0; slice gap 0.6 mm); transversal T2-weigted turbo spin echo sequence (TR/TE 7,440/96 ms; voxel size 0.9/0.9 × 3.0 mm; slice gap 0.6 mm); and gradient echo susceptibility-weighted imaging (SWI) sequence (TR/TE 49/40 ms; voxel size 0.8 × 0.7 × 1.0 mm). All patients underwent MRI scanning according to this standardized protocol.^8^

### Small vessel disease.

SVD was defined according to the Standards for Reporting Vascular Changes on Neuroimaging criteria.^15^ WMHs of presumed vascular origin were defined as hyperintense signal abnormalities in the white matter on FLAIR images, without cavitation.^15^ Hyperintensities in the subcortical gray matter or brainstem were also included in the analysis. Gliosis surrounding lacunar and territorial infarcts was not considered to be WMHs. WMH volumes were determined using a validated in-house developed fully automated method.^16^ This computer-aided detection system uses a supervised machine learning approach being trained on a dataset of more than 300 MRI with WMHs annotated on them. More than 20 features were utilized to describe for each voxel the intensities of FLAIR and T1 modalities, location, and the shape of the structure each voxel belongs to. The system has been evaluated on an independent dataset of 32 images and it was shown to be performing close to a human observer. All scans were checked by visual inspection. WMH volumes were normalized to intracranial volume (ICV). Voxel-based morphometry toolbox within SPM8 was used for each T1 image to determine the volume of gray matter, white matter, and CSF, in order to calculate ICV.^17^ In addition, WMHs were manually rated according to the Fazekas score: 0 = 1 or no hyperintensities, 1 = focal hyperintensities, 2 = beginning confluence, and 3 = confluent hyperintensities with diffuse involvement of the entire region.^18^

Lacunes of presumed vascular origin were defined as round or ovoid, subcortical, fluid-filled cavities, of between 3 mm and about 15 mm in diameter, consistent with a previous acute small deep brain infarct in the territory of 1 perforating arteriole.^15^

Cerebral microbleeds were defined as small areas less than 10 mm in diameter of signal void with associated blooming seen on SWI.^15^ Signal voids in areas of territorial infarcts were not considered to be microbleeds. For each participant, the presence and numbers of lacunes and all MRI scans were analyzed by a trained rater blinded to clinical and demographic data. In a random sample of 10%, the interrater reliability for the presence of lacunes yielded a kappa of 0.76 and intrarater reliability yielded a kappa of 0.80. For the Fazekas score, interrater and intrarater reliability yielded a weighted kappa of 0.68 and 0.88, respectively. Interrater and intrarater reliability for the presence of microbleeds yielded a kappa of 1.0 and 0.92, respectively.

### Vascular risk factors at follow-up.

At follow-up, blood pressure was measured in supine position 3 times on both arms, of which the highest measurement was used to define hypertension. Hypertension was defined as systolic blood pressure ≥135 mm Hg or diastolic blood pressure ≥85 mm Hg^19^ or the use of antihypertensive medication. Hypertension was divided into 3 categories: treated but uncontrolled hypertension, hypertension controlled with medication, and untreated hypertension. Diabetes mellitus (DM) was defined as random blood glucose level >11.1 mmol/L or 2 consecutive fasting venous plasma glucose levels ≥7.0 mmol/L^20^ or the use of antidiabetics (oral or insulin). Dyslipidemia was defined as total cholesterol ≥5.0 mmol/L or low-density lipoprotein ≥2.5 mmol/L or triglycerides ≥2.0 mmol/L^21^ or the use of statins. Whenever blood pressure, glucose levels, or cholesterol levels were missing (0.2%, 2.3%, and 3.3%, respectively), a medical history of DM, dyslipidemia, or hypertension was used. In addition, information on smoking was collected by a structured questionnaire. Current smoking was defined as smoking ≥1 cigarette per day in the year prior to follow-up (0.5% missing).

### Statistical analysis.

Baseline characteristics and the prevalence of SVD between patients and controls were compared using χ^2^, Student *t* test, or Mann-Whitney *U* whenever appropriate. In addition, the prevalence of lacunes, microbleeds, and mean WMH volume was stratified by age at MRI (20–39, 40–54, and 55–79 years).

By means of binary logistic regression, the prevalence of lacunes and microbleeds were compared between patients and controls by calculating odds ratios (ORs) with their 95% confidence intervals (CIs). Linear regression was used to compare WMH volume between patients and controls, by calculating βs. WMH volumes were log transformed because of skewness of the untransformed measure. As the data contained zeros (n = 21), which cannot be log transformed, a constant number of 0.001 (which equaled the smallest volume in the dataset) was added to all data before transformation. Confounders in the logistic regression and linear regression model were age, sex, smoking, DM, and hypertension at follow-up.

Within the patient group, by means of binary logistic regression and linear regression, we calculated ORs and βs of individual baseline variables with 95% CIs for the risk of lacunes, microbleeds, and WMH volume, respectively. Each baseline risk factor was only adjusted for sex, age at event, and follow-up duration. In addition, for WMH volume, independent βs with 95% CIs were calculated for vascular risk factors using the enter method. Variables in the model were sex, age at stroke, follow-up duration, hypertension, DM, and smoking.

Finally, to compare WMH volume between patients and controls, mean WMH volume was stratified by age groups of 5 years. To investigate the vulnerability of the brain to aging, the relation between age and WMH volume was investigated for patients and controls separately. WMH volume seemed to be exponential related to age (*R*^2^ = 0.97) for patients and polynomial for controls (*R*^2^ = 0.96). We then calculated group differences in WMH volume to establish estimates of the number of years of age necessary for controls to achieve the same mean WMH volume value as patients.

SPSS 20 (Chicago, IL) was used for all statistical analyses.

## RESULTS

### Study population.

A total of 337 patients (110 with a TIA, 227 with an ischemic stroke) and 90 controls were included in the present study (figure e-1 at Neurology.org). Table e-1 shows baseline characteristics for patients who participated in the present study and those who did not. Nonparticipants less often had an undetermined cause of their stroke (31.9% vs 40.9%) and more often were smokers (55.3% vs 45.3%).

Mean age at index event was 40.0 years (SD 7.9) and 45.4% of them were men. After a mean follow-up of 9.9 years (SD 8.1), mean age was 49.8 years (SD 10.3) for patients and 49.4 years (SD 11.9) for controls. Baseline and follow-up characteristics are shown in table 1.

**Table 1:**
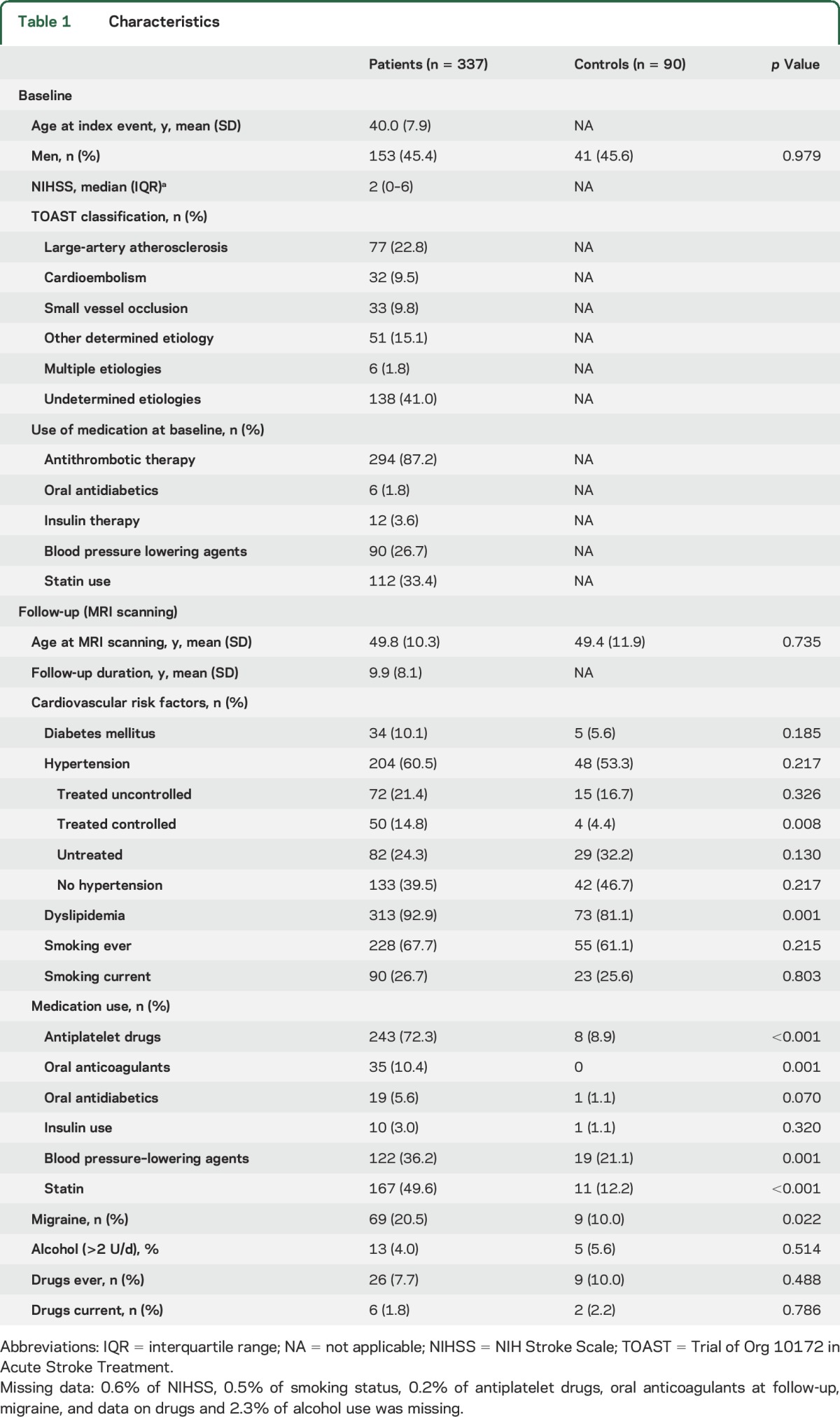
Characteristics

### Stroke in young adults vs controls.

After mean follow-up of 9.9 years (SD 8.1), 81 patients (24.0%) had at least 1 lacune vs 4 controls (4.5%) (*p* < 0.001). Forty-four (13.1%) There was no difference in microbleeds between patients and controls (n = 44, 13.1% and n = 6, 6.7% respectively). Patients had a higher WMH volume than controls (median 1.5 mL [interquartile range (IQR) 0.5–3.7] vs 0.4 mL [IQR 0.0–1.0], *p* < 0.001) (table 2).

**Table 2:**
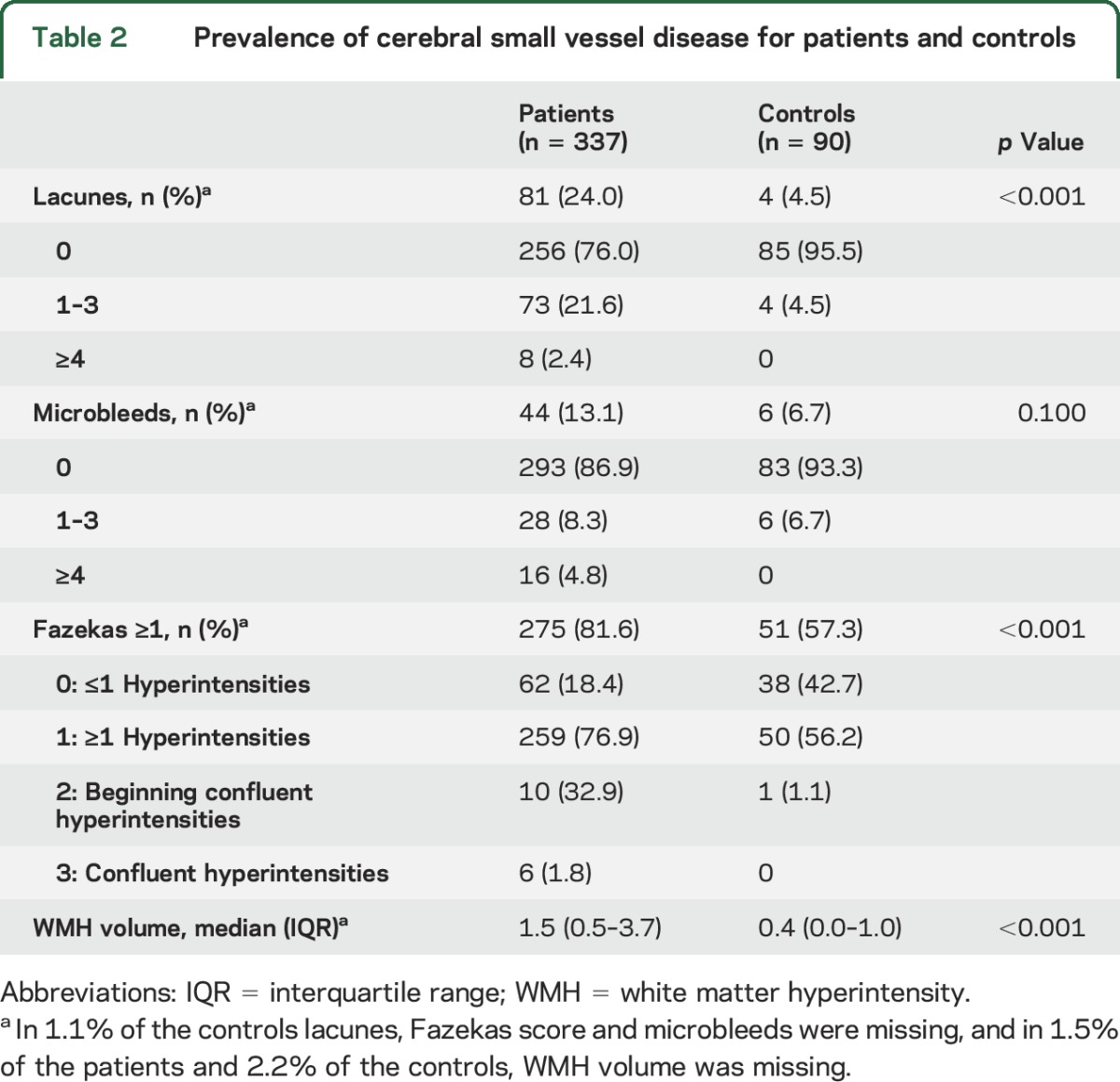
Prevalence of cerebral small vessel disease for patients and controls

Logistic regression showed that patients remained at a higher risk for lacunes than controls after adjustment for confounders (age, sex, smoking, DM, and hypertension at follow-up) (OR 6.8, 95% CI 2.4–19.8, *p* < 0.001). Linear regression showed a higher WMH volume for patients compared to controls after adjustment for confounders (β = 0.82, 95% CI 0.63–1.01, *p* < 0.001). Table 3 shows the prevalence of lacunes and microbleeds and WMH volume for patients and controls stratified by age at follow-up.

**Table 3:**
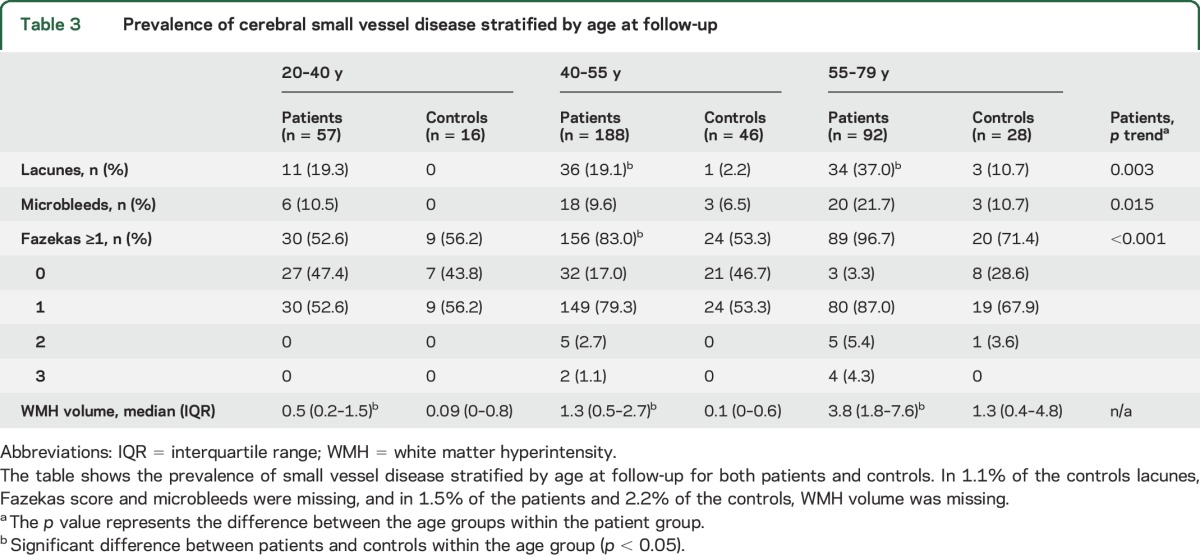
Prevalence of cerebral small vessel disease stratified by age at follow-up

For patients, the prevalence of microbleeds and lacunes and the volume of WMHs increased by age. In addition, beginning confluence and confluent WMHs were not present in patients aged 20–40 years, in contrast to the higher age groups. In all age groups, WMH volume was higher among patients than among controls. In addition, lacunes were more prevalent among patients compared to controls in the age groups 40–55 years and 55–79 years and not in the youngest age group.

**Figure 1:**
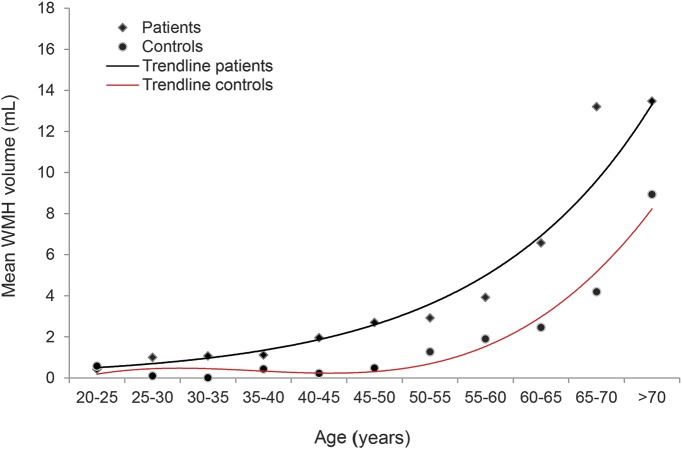
Relation between age at follow-up and white matter hyperintensity (WMH) volume for patients and controls Mean WMH volume stratified by age groups of 5 years.

Figure 1 shows the relation between age at follow-up and WMH volume both for patients and controls. Compared with controls, patients had the same volume of WMH on average 10–20 years earlier (figure e-2).

### Baseline risk factors for SVD in patients with stroke at young age.

Baseline risk factors associated with microbleeds were male sex (OR 2.0, 95% CI 1.0–3.9, *p* = 0.043) and hypertension (OR 1.1, 95% CI 1.0–1.1 *p* = 0.009). Male sex was also associated with lacunes (OR 2.3, 95% CI 1.3–3.9, *p* = 0.002). Age at stroke (β = 0.03, 95% CI 0.02–0.04, *p* < 0.001), hypertension (β = 0.22, 95% CI 0.04–0.39), and smoking (β = 0.18, 95% CI 0.01–0.34, *p* = 0.035) were associated with WMH volume (table 4).

**Table 4:**
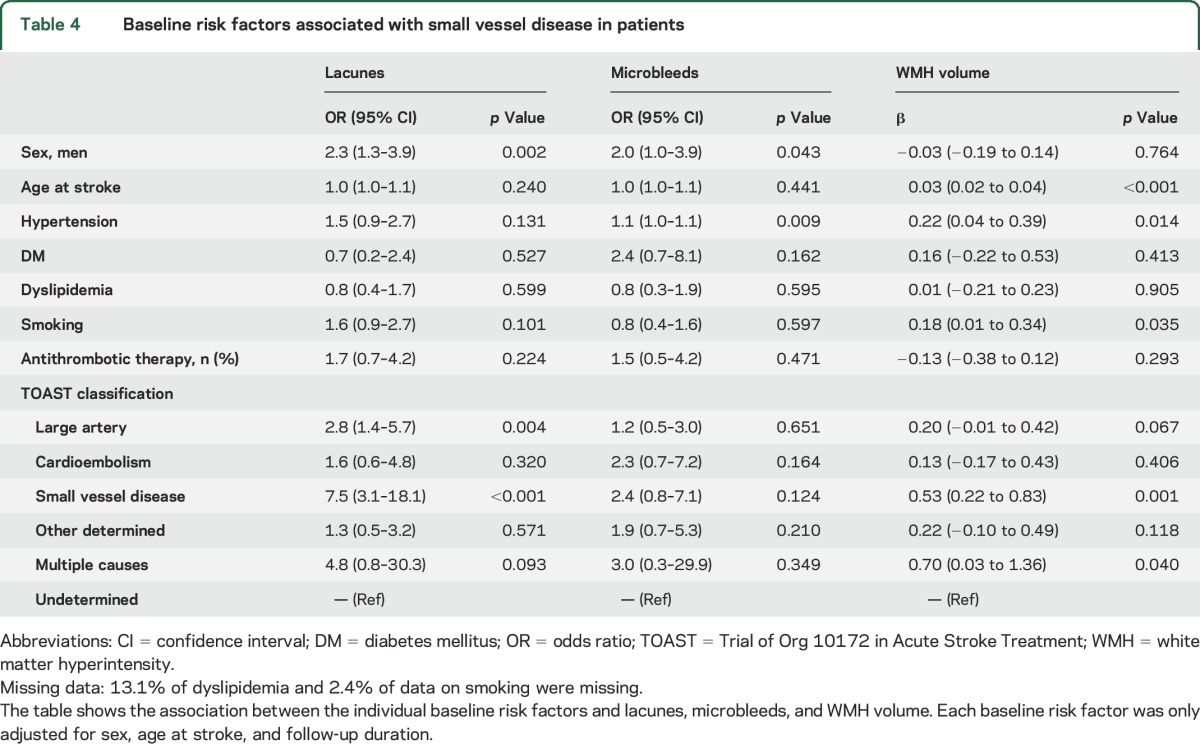
Baseline risk factors associated with small vessel disease in patients

In addition, age at stroke (β = 0.03, 95% CI 0.02–0.04, *p* < 0.001), follow-up duration (β = 0.04, 95% CI 0.03–0.05, *p* < 0.004), and hypertension (β = 0.20, 95% CI 0.02–0.38, *p* = 0.029), were independently associated with WMH volume adjusted for sex, DM, and smoking (*R*^2^ = 0.23).

## DISCUSSION

We have shown that on average after 10 years of follow-up of patients with a stroke at young age, patients have a higher burden of lacunes and WMHs than controls who did not experience a stroke at young age but had otherwise identical vascular risk factors. In addition, patients already had the same volume of WMHs on average 10–20 years earlier in life than controls.

Strengths of our study include the large population of patients with a stroke at young age and the single-center design, which made it possible to collect all data systematically and uniformly in all participants.^19^ In addition, a trained rater who analyzed the MRI scans was blinded to clinical and demographic data, making information bias less likely. Furthermore, the inclusion of a control group made it possible to compare the prevalence of cerebral SVD between patients with a stroke at young age and controls.

However, some limitations in our study need to be considered. First, it may be possible that selection bias occurred in our study due to selective loss to follow-up. Patients who did not participate in the present substudy more often were smokers compared to participants. Since smoking was associated with WMH volume, this may have led to an underestimation of WMH volume in patients. Second, baseline neuroimaging was performed according to different clinical routines, for example, due to the long inclusion period; therefore we were not able to determine incident SVD, but only its prevalence, which made it a cross-sectional study. Third, confounding may have influenced our results, but we have tried to overcome this by adjusting for the most important confounders for SVD.

We have shown that patients with a stroke at young age have a significantly higher burden of lacunes and WMHs than controls, even after adjustment for confounders. Age at follow-up was an important factor associated with SVD. We found a higher prevalence of WMHs than 2 retrospective studies in patients with a stroke at young age, which reported a prevalence of WMHs of around 7%.^22,23^ Another study on MRI characteristics in patients with a stroke at young age also reported a lower prevalence of 45% WMHs and 7% microbleeds.^24^ However, MRI in those studies was performed during the initial workup directly after the index stroke. Due to our longer follow-up, patients were older at the time of the MRI scan and had a higher prevalence of vascular risk factors. In addition, WMHs in those studies was only rated by the Fazekas score and not quantitative. Due to the combination of a high prevalence of vascular risk factors and long follow-up duration, patients may have been exposed to those risk factors for a longer period. However, 10 years after the stroke, patients in our study still were young and had a mean age of only 49.8 years and the prevalence of vascular risk factors was identical in our controls.

An explanation for the high prevalence of SVD among stroke in young adults may be that patients are, for unknown reasons, more vulnerable to those vascular risk factors than others. A possible explanation might be that there is a genetic predisposition for developing cardiovascular diseases; however, this has not been investigated. Another possibility might be that patients already have been exposed to these vascular risk factors for a longer period than controls. For example, it has been shown that blood pressure is associated with subtle vascular brain injury such as reduced cerebral integrity already very early in life.^25^

Another explanation might be that patients already had SVD due to their initial stroke. However, in only 10% was the etiology of the index event SVD, which is comparably low to other studies on stroke in young adults and makes this an unlikely explanation.^26,27^ With respect to the TOAST classification, patients with SVD as the cause of their initial stroke showed the most pronounced association compared to undetermined etiology with the presence of lacunes and increased WMH volume. This has also been found in elderly populations.^28^ Strokes due to large artery disease and multiple causes also were associated with the presence of lacunes and increased WMH volume. An explanation might be that large artery disease and SVD share underlying vascular risk factors, including age at stroke and smoking, which were associated with WMH volume in the present study.

In addition, besides having a higher burden of SVD, another striking finding was that patients already had on average 10–20 years earlier in life the same volume of WMHs as controls who have an identical traditional vascular risk profile. This may be an additional argument that these patients are very vulnerable to the deleterious effects of vascular risk factors decades before their healthy peers. The suggested increased vulnerability supports the need for accurate identification of vascular risk factors at presentation and immediate treatment accordingly, even in these often young patients. Although this has never been investigated, it may be an argument for lifelong secondary prevention strategies.

Apart from gaining more insight into possible etiologic mechanisms, the occurrence of SVD may also have prognostic implications. In elderly patients, SVD has been associated with depression and cognitive impairments,^5–7^ independent of stroke. A large MRI study in patients younger than 55 with a stroke found no association between depression and MRI characteristics of SVD.^29^ However, both MRI and assessment of depression took place within 10 days after stroke. Patients may develop both SVD and depression during follow-up, which determines poststroke functioning. Especially in young patients, this long-term follow-up is of utmost importance.

We have shown that SVD is very prevalent in patients with a stroke at young age and is associated with age at follow-up. Cerebral aging seems accelerated by 10–20 years in these patients, which may indicate an increased vulnerability to vascular risk factors.

## Supplementary Material

Data Supplement

## GLOSSARY

CI: confidence interval
DM: diabetes mellitus
FLAIR: fluid-attenuated inversion recovery
FUTURE: Follow-Up of Transient Ischemic Attack and Stroke Patients and Unelucidated Risk Factor Evaluation
ICV: intracranial volume
IQR: interquartile range
OR: odds ratio
SVD: small vessel disease
SWI: susceptibility-weighted imaging
TE: echo time
TI: inversion time
TOAST: Trial of Org 10172 in Acute Stroke Treatment
TR: repetition time
WMH: white matter hyperintensity
