# Supplementary material for: Accelerated development of cerebral small vessel disease in young stroke patients
Source: Neurology. 2016 Sep 20;87(12):1212–9. doi: 10.1212/WNL.0000000000003123 (PMC5035980; doi:10.1212/WNL.0000000000003123)
Supplement: Data Supplement [file supp_87_12_1212__index.html]

Accelerated development of cerebral small vessel disease in young stroke patients — Data Supplement 

# Accelerated development of cerebral small vessel disease in young stroke patients

## Data Supplement

**Neurology® data supplements are not copyedited before publication. Published editorials and translations have been copyedited.  
 © 2016 American Academy of Neurology.  
  
 Files in this Data Supplement:**

- Data Supplement - Microsoft Word file
