# Supplementary material for: Accelerated development of cerebral small vessel disease in young stroke patients
Source: Neurology. 2016 Sep 20;87(12):1212–9. doi: 10.1212/WNL.0000000000003123 (PMC5035980; doi:10.1212/WNL.0000000000003123)
Supplement: Data Supplement [file supp_WNL.0000000000003123_Supplementary_files.docx]

Supplementary files

Figure e-1 Flowchart patients selection

Table e-1 Baseline characteristics for patients included and excluded in the present study

|  | | Included in present study (n=337) | Excluded in present study^a^ (n=386) | p-value |
| --- | --- | --- | --- | --- |
| Index event, n (%) | |  |  |  |
|  | TIA | 110 (32.6) | 129 (33.4) |  |
|  | Ischemic stroke | 227 (67.4) | 257 (66.6) | 0.824 |
| Age at index event, mean (SD) | | 40.0 (7.9) | 39.9 (8.0) | 0.617 |
| Men, n (%) | | 153 (45.4) | 166 (43.0) | 0.518 |
| NIHSS, median (IQR) | | 2 (0-6) | 2 (1-6) | 0.195 |
| TOAST-classification | |  |  |  |
|  | Large-artery atherosclerosis | 77 (22.8) | 93 (24.1) | 0.694 |
|  | Cardio-embolism | 32 (9.5) | 48 (12.4) | 0.209 |
|  | Small vessel occlusion | 33 (9.8) | 48 (12.4) | 0.261 |
|  | Other determined etiology | 51 (15.1) | 66 (17.1) | 0.474 |
|  | Multiple etiologies | 6 (1.8) | 8 (2.1) | 0.776 |
|  | Undetermined etiology | 138 (40.9) | 123 (31.9) | 0.011 |
| Diabetes mellitus | | 16 (4.7) | 23 (6.0) | 0.472 |
| Hypertension | | 97 (28.8) | 118 (30.6) | 0.600 |
| Dyslipidemia^b^ | | 232 (68.8) | 252 (65.3) | 0.984 |
| Smoking^b^ | | 149 (45.3) | 208 (55.3) | 0.008 |

^a^ Patients who refused to participate, were lost to follow-up or did not participate in the MRI sub study.

^b^ information on NIHSS was missing in 0.6%, information on dyslipidemia in 15.5%, and smoking status in 2.5%

Figure e-2 Stroke related brain ageing

This figure shows the difference in age at which both controls and patients had identical WMH volumes (Y-axis) and on the X-axis the age of the patient at follow up.

Volumes of WMH were calculated for both patients and controls by age. The curved line indicates the difference in age between controls and patients at which both patients and controls have exactly the same mean WMH-volume. For example, the dotted line indicates WMH-volume of a 50 year old patient with a stroke at young age, which corresponds to WMH-volume of a control who is 12.8 years older (*ie* 62.8 years).
